# Lung Isolation in a Child with Kinsbourne Syndrome for Paraspinal Neuroblastoma Excision in the Prone Position

**DOI:** 10.4274/TJAR.2025.251960

**Published:** 2025-12-22

**Authors:** Aritra Kundu, Nishant Patel, Subodh Kumar, Rakesh Kumar, Sachin Kumar, Vishesh Jain

**Affiliations:** 1 All India Institute of Medical Sciences, Department of Anaesthesiology, Pain Medicine & Critical Care, New Delhi, India; 2 All India Institute of Medical Sciences, Department of Paediatric Surgery, New Delhi, India

**Keywords:** Airway management, cardiovascular and thoracic anaesthesia, paediatric anaesthesia, perioperative care

## Abstract

Kinsbourne syndrome, also known asor opsoclonus-myoclonus-ataxia syndrome, is a rare paediatric neurological disorder characterised by abnormal eye movements, myoclonus, and ataxia. Its anaesthetic management presents significant challenges, especially when one-lung ventilation (OLV) is required in the prone position. This case report describes the anaesthetic management of a two year-old child with Kinsbourne syndrome undergoing T9-T11 paravertebral neuroblastoma excision. Because of the patient’s size and the need for lung isolation, a Fogarty embolectomy catheter was used for OLV. Anaesthesia was induced with intravenous fentanyl, propofol, and atracurium, followed by the insertion of a 4.0 mm cuffed endotracheal tube to facilitate Fogarty catheter insertion. The catheter was positioned in the right bronchus under fibre-optic guidance; after which, a 4.5 mm cuffed tube was inserted, and the patient was placed in the prone position. Continuous fibre-optic monitoring ensured proper catheter placement. Anaesthesia was maintained with oxygen, air, and isoflurane. The patient remained haemodynamically stable, was extubated postoperatively, was observed in the paediatric intensive care unit for 24 hours, and was subsequently transferred to the ward. This case highlights the challenges of OLV in paediatric patients and demonstrates the effectiveness of a Fogarty catheter for lung isolation when traditional devices are unsuitable, emphasising the importance of multidisciplinary collaboration and continuous monitoring.

**Main Points**

• Complexity of Anaesthesia in Opsoclonus-myoclonus-ataxia (OMA) with Neuroblastoma: OMA (Kinsbourne) syndrome associated with paraspinal neuroblastoma presents unique perioperative challenges, particularly regarding airway and respiratory management.

• Lung Isolation in Small Children: Standard double-lumen tubes are unsuitable in infants/toddlers; a Fogarty embolectomy catheter proved an effective alternative for one-lung ventilation (OLV), allowing safe tumour resection in the prone position.

• Challenges of OLV in Paediatrics: Risks of hypoxia, hypercapnia, airway trauma, and catheter migration necessitate fibre-optic-guided placement and continuous intraoperative monitoring.

• Fogarty catheters can be a practical and safe solution for paediatric lung isolation, provided multidisciplinary coordination, fibre-optic confirmation, and vigilant monitoring are maintained.

## Introduction

Neuroblastoma is a common extracranial solid tumour in paediatric patients, typically originating in the adrenal medulla or paraspinal ganglia. Surgical resection often requires specific anaesthetic considerations, mainly when the lesion involves the thoracic region. The presence of opsoclonus-myoclonus-ataxia syndrome (OMA), also known as Kinsbourne syndrome, a paraneoplastic condition associated with neuroblastoma, further complicates the perioperative management. This case report presents the anaesthetic approach for a two-year-old male child with neuroblastoma at the T9-T11 level, highlighting lung isolation using a Fogarty embolectomy catheter in the prone position, an uncommon yet necessary technique for the resection of paraspinal tumours.

## Case Report

A two-year-old male patient (height/weight: 86 cm/11.5 kg), diagnosed with a T9-T11 paravertebral neuroblastoma associated with OMA, was scheduled for tumour resection. The pre-operative assessment revealed a normal cardiorespiratory status and mild ataxia, with no other systemic involvement. Due to the tumours location, a thoracic approach with lung isolation was deemed necessary to provide adequate surgical exposure and prevent contamination of the contralateral lung. Informed written parental consent for anaesthesia was obtained.

### Anaesthesia Plan

**Induction:** Anaesthesia was induced with intravenous fentanyl (2 µg kg^-1^), propofol (2 mg kg^-1^), and atracurium (0.5 mg kg^-1^) to achieve muscle relaxation. Subsequently, the patient was intubated with an appropriately sized #4.0 cuffed endotracheal tube.

**Lung Isolation:** Initially, a 4 French Fogarty embolectomy catheter was passed through the #4.0 endotracheal tube until resistance was encountered that prevented further advancement of the catheter. The cuffed endotracheal tube was then deflated and removed. Another #4.5 cuffed tube was subsequently introduced under fibre-optic guidance, until it reached the vocal cords. The fiber-optic was further introduced to visualise the carina and right bronchus, facilitating precise positioning of the Fogarty catheter. The catheter was placed in the right bronchus, and the cuff was inflated with 1 mL of air, confirming the isolation of the right lung. This technique was selected due to the patient’s small size, which made the use of double-lumen endotracheal tubes unfeasible, and the need for selective lung isolation while the patient was in the prone position.

**Figure 1:**
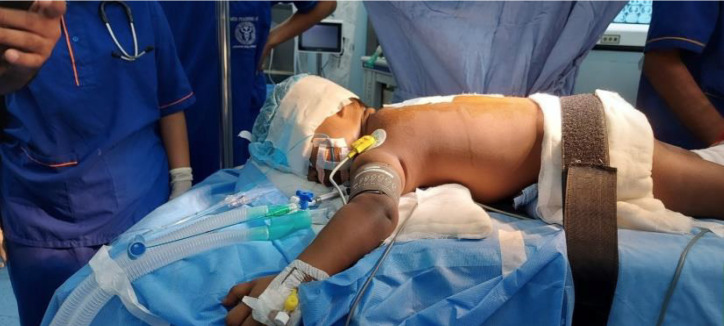
Positioning with Forgarty catheter in prone position.

**Positioning:** The patient was placed in the prone position, with careful padding of pressure points and maintenance of neutral alignment of the head and neck. Continuous monitoring of airway pressures and oxygenation was crucial, given the potential difficulties associated with one-lung ventilation (OLV) in this age group (Figure 1).

**Maintenance:** Anaesthesia was maintained using a mixture of oxygen (50%), nitrous oxide (50%), and isoflurane (1%). Muscle relaxation was achieved through intermittent doses of atracurium. Monitoring included end-tidal carbon dioxide (CO_2_), pulse oximetry, and arterial blood gas analysis to ensure adequate ventilation and oxygenation during OLV.

### Intraoperative Course

The patient remained haemodynamically stable throughout the procedure. The use of the Fogarty catheter allowed successful isolation of the right lung, ensuring adequate visualisation of the tumour without compromising ventilation. Blood gas levels remained within acceptable limits, with only a minimal increase in partial pressure of CO_2_ in arterial blood. No complications were observed related to positioning, and the surgery proceeded as planned, culminating in the complete resection of the tumour.

### Postoperative Management

Postoperatively, the patient was extubated in the operating room following the return of adequate spontaneous ventilation and neuromuscular function. He was closely monitored in the paediatric intensive care unit (PICU) for 24 hours due to concerns regarding respiratory compromise and the potential effects of OMA. No postoperative complications were noted, and the patient was discharged from the PICU on postoperative day 2.

## Discussion

The anaesthetic management of neuroblastoma resection, particularly in the presence of OMA, poses multiple challenges, notably in airway management, patient positioning, and the risk of respiratory complications. Lung isolation techniques in paediatric patients differ significantly from those used in adults due to anatomical limitations, including narrower airways and greater susceptibility to airway trauma. Traditional methods, such as double-lumen tubes, are unsuitable for infants and young children. As demonstrated in this case, the Fogarty embolectomy catheter serves as an effective alternative for achieving lung isolation in paediatric patients, offering controlled ventilation to one lung while providing an unobstructed surgical field.^1^

OLV in paediatric patients, particularly in infants and toddlers, presents several challenges, including hypoxia and hypercapnia. Achieving effective lung isolation with minimal airway trauma is critical, particularly when prone positioning is utilised. Various techniques for lung isolation include the use of bronchial blockers, such as the Arndt, Cohen, or Fogarty embolectomy catheters, as employed in this case. The appropriate device selection is contingent upon the child’s age, size, and surgical requirements.^2^

The most commonly used techniques for lung isolation in children include:

1. Endobronchial intubation involves using a single-lumen endotracheal tube pushed into one of the mainstem bronchi. This technique is simple and widely used in younger children; however, it lacks precision. It may result in unintentional movement, particularly during patient repositioning, as was observed in the present case.^3^

2. Bronchial blockers, such as the Arndt or Cohen blockers, are frequently employed in paediatric patients, allowing for selective occlusion of one lung while ventilating the other. However, their placement can be technically demanding in younger children and may necessitate additional equipment such as fibre-optic bronchoscopes for accurate positioning. Additionally, in infants, these blockers can increase airway resistance, complicating the maintenance of adequate ventilation during OLV.^3, 4^

3. Fogarty embolectomy catheters, as used in this case, offer a viable alternative for small paediatric patients. Their small size and ease of insertion make them suitable when bronchial blockers or double-lumen tubes are impractical. Nevertheless, the primary concern with this technique is the potential for catheter migration or airway obstruction, requiring continuous monitoring and fibre-optic confirmation of placement throughout the surgery.^5, 6^

### Concerns Regarding Prone Positioning and OLV

Prone positioning introduces additional challenges in managing OLV, as it significantly changes ventilation mechanics due to altered chest wall compliance and intra-abdominal pressure.^7^

### These changes may lead to:

**1. Increased airway pressures:** The prone position can elevate airway pressures under OLV, primarily due to decreased compliance of the dependent lung and mechanical compression of the chest wall by the operating table.

**2. Ventilation-perfusion mismatch:** In the prone position, ventilation is directed towards the non-dependent lung, which, during OLV, becomes the sole ventilated lung. This shift can cause significant ventilation-perfusion mismatch, potentially leading to hypoxia, especially in younger patients with a smaller functional residual capacity.^7, 8^

**3. Haemodynamic changes:** The combination of the prone position and OLV can reduce venous return due to the compression of abdominal organs and large vessels. This reduction may lead to hypotension, further impairing oxygen delivery. Therefore, maintaining adequate intravascular volume and frequent monitoring of haemodynamics are crucial.

**4. Limited access to airway management:** A major concern with prone positioning during OLV is restricted access to the airway. If the lung isolation device becomes dislodged or airway obstruction occurs, repositioning and reintubation can be challenging. This risk necessitates continuous vigilance and the availability of fibre-optic bronchoscopy equipment to ensure proper placement of the catheter or bronchial blocker.

**5. Postoperative complications:** Prone positioning with OLV increases the risk of atelectasis in the non-ventilated lung, particularly if recruitment manoeuvers or adequate postoperative physiotherapy are not performed. In paediatric patients, these postoperative respiratory complications may be more pronounced, necessitating meticulous postoperative care, including respiratory support.^9^

In this case, careful consideration of the patient’s size and the need for prone positioning led to the selection of the Fogarty catheter for lung isolation. The catheter placement was confirmed via fibre-optic bronchoscopy, while close monitoring of ventilator parameters ensured that the patient tolerated OLV effectively despite the challenges linked to prone positioning.

## Conclusion

This case highlights the complexities of managing paediatric patients requiring lung isolation for neuroblastoma resection, particularly in the prone position. Effective lung isolation techniques, such as Fogarty embolectomy catheters, can be successfully implemented in young children. However, careful attention must be paid to the complications associated with prone positioning and OLV, including increased airway pressures, ventilation-perfusion mismatch, and haemodynamic instability. Continuous monitoring, alongside the availability of fibre-optic equipment, is vital to ensure safe and effective anaesthetic management during these challenging procedures.

## Ethics

**Informed Consent:** Informed written parental consent for anaesthesia was obtained.
